# Cancer as a moving target: understanding the composition and rebound growth kinetics of recurrent tumors

**DOI:** 10.1111/eva.12019

**Published:** 2012-11-16

**Authors:** Jasmine Foo, Kevin Leder, Shannon M Mumenthaler

**Affiliations:** 1 School of Mathematics, University of MinnesotaMinneapolis, MN, USA; 2 Industrial and Systems Engineering, University of MinnesotaMinneapolis, MN, USA; 3 Center for Applied Molecular Medicine, University of Southern California Keck School of MedicineLos Angeles, CA, USA

**Keywords:** biomedicine, evolutionary theory, population genetics – theoretical

## Abstract

We introduce a stochastic branching process model of diversity in recurrent tumors whose growth is driven by drug resistance. Here, an initially declining population can escape certain extinction via the production of mutants whose fitness is drawn at random from a mutational fitness landscape. Using a combination of analytical and computational techniques, we study the rebound growth kinetics and composition of the relapsed tumor. We find that the diversity of relapsed tumors is strongly affected by the shape of the mutational fitness distribution. Interestingly, the model exhibits a qualitative shift in behavior depending on the balance between mutation rate and initial population size. In high mutation settings, recurrence timing is a strong predictor of the diversity of the relapsed tumor, whereas in the low mutation rate regime, recurrence timing is a good predictor of tumor aggressiveness. Analysis reveals that in the high mutation regime, stochasticity in recurrence timing is driven by the random survival of small resistant populations rather than variability in production of resistance from the sensitive population, whereas the opposite is true in the low mutation rate setting. These conclusions contribute to an evolutionary understanding of the suitability of tumor size and time of recurrence as prognostic and predictive factors in cancer.

Despite the initial effectiveness of many anticancer therapies in reducing tumor size and halting growth, many tumors eventually resume growth after a period of time during therapy due to the evolution of drug-resistant clones. In recent work, Ding et al. (2012) observed clonal evolution in relapsed acute myeloid leukemia (AML) using whole genome sequencing. By sequencing the primary tumor and relapse genomes from AML patients, they observed that while some tumor subclones are indeed eradicated by therapy, others accumulate new mutations and subsequently expand during cancer recurrence. As a result, relapsed or recurrent tumors can be highly heterogeneous in nature, and their composition can differ significantly from that of the original tumor. These observations, part of a growing literature documenting clonal evolution of the cancer genome (the review of Merlo et al. 2006 and references therein), lend credence to the idea that *cancer genomes are moving targets*. This suggests that targeting only cell types present at the start of therapy is insufficient to eradicate tumors, and furthermore, therapy may itself impact or enhance the clonal evolution of resistant subpopulations.

An understanding of the amount of clonal diversity present in recurrent tumors driven by drug-resistant cell populations is important for determining optimal treatment strategies after failure of a first-line therapy. However, due to limitations in detecting mutations in rare cells, experimental studies may provide only an estimate of the lower bound on clonal heterogeneity in recurrent tumors. Here, we aim to obtain a better understanding of the major factors impacting the composition and growth of heterogeneous recurrent cancer cell populations using evolutionary modeling. We study how, after an initial decline in tumor size, the rebound growth kinetics and composition of the recurrent tumor are affected by evolutionary parameters such as the fitness landscape of mutations accumulated during therapy, initial size, drug effectiveness, and mutation rates. In addition, we derive estimates of the amount of clonal diversity present in relapsed tumors and demonstrate a strong dependence on the shape of the mutational fitness landscape. We also study the relationship between the timing of cancer recurrence and the diversity and aggressiveness of the relapsed tumor.

There has been a significant amount of research interest in evolutionary modeling of drug resistance in cancer, both prior to treatment in expanding tumor cell populations as well as during treatment. For example, Coldman and collaborators introduced stochastic models of the emergence of resistance to chemotherapy (Goldie and Coldman 1979, 1983; Coldman and Goldie 1986; Coldman and Murray 2000) to guide treatment schedules. Jackson and Byrne (2000) considered a deterministic PDE model describing the intra-tumoral drug concentration and density of sensitive and resistant cancer cells and investigated the tumor response to continuous infusion versus bolus injection of chemotherapeutic drugs in the presence and absence of drug-resistant subpopulations. Komarova and Wodarz (2005, 2007) developed a model for multi-drug resistance using a multi-type birth–death process in which the resistance to each drug was conferred by genetic alterations within a mutational network. Iwasa et al. (2003), Michor et al. (2006) and Haeno et al. (2007) studied the dynamics of resistance emerging due to one or two genetic alterations in a clonally expanding population of sensitive cancer cells. More recently, Silva and Gatenby (2010) introduced a model that incorporated the interactions of cell resistance mechanisms and tumor microenvironment during chemotherapy and found that a combined treatment strategy of glucose restriction and chemotherapy can stabilize tumor size and minimize resistant populations. These references represent a few examples from a large and growing literature of evolutionary models of drug resistance in cancer.

Most of the existing mathematical formulations described consider cells with identical genotypes to have identical fitness characteristics. However, single-cell profiling studies have revealed extraordinary heterogeneity in phenotype even if genetically, these cells are identical (Elowitz et al. 2002; Kaern et al. 2005; Feinerman et al. 2008). In particular, variable fitness effects have been observed in cancer cell lines harboring the same drug-resistance mechanisms (Godin-Heymann et al. 2007; Ohashi and et al. 2012). For example, Godin-Heymann et al. (2007) produced data exhibiting approximately 10% variation in growth rates between non-small cell lung cancer clones harboring the same resistance mechanism to the drug erlotinib. Alternatively, there may exist a spectrum of distinct possible resistance mutations for a single drug, each conferring a different response to therapy (Sierra et al. 2010; Xu et al. 2012). For example, in chronic myeloid leukemia (CML), resistance to the tyrosine kinase inhibitor imatinib can be conferred by over 90 distinct resistance mutations, and these mutations confer different fitness advantages or disadvantages relative to the unmutated drug-sensitive cells (Skaggs et al. 2006; Sierra et al. 2010; Leder et al. 2011). In considering 11 of the most common resistant mutants in CML, Skaggs et al. (2006) determined that differences in relative growth rates *in vitro* between resistant mutants can exhibit as much as 30% variation above and below the average.

Therefore, it is important to develop a quantitative understanding of the diversity of heterogeneous drug-resistant cancer cell populations that drive resistance. In recent work, we introduced a model of the evolution of heterogeneity during tumorigenesis, describing the accumulation of combinations of mutations that confer random alterations to cellular fitness in an exponentially expanding population (Durrett et al. 2010, 2011). In the current work, we consider a different problem in which escape from inevitable extinction of the initial population occurs via the generation of diverse mutant populations. Here, we adopt a modified mathematical framework and perform analysis in a different asymptotic regime (of large initial population size) to study the properties of relapsed tumors after an initial response during treatment. In other recent work (Foo and Leder 2012), we examined the probability distribution of recurrence times in a simple model of homogeneous escape populations; here, we focus on the composition and diversity of heterogeneous escape populations and explore the relationship between recurrence timing and composition of the relapsed tumor.

The paper is outlined as follows. In the Model section, we introduce the model and relevant notations to be used in the paper. We also provide some sample simulations to illustrate the diversity in the rebound population and variability in recurrence timing. In Results section, we establish analytical results regarding the rebound growth kinetics of the heterogeneous tumor after relapse. Then, we investigate the composition and diversity of the relapsed tumor and study the relationship between recurrence time and diversity of the relapsed tumor.

## Model

In the following, we consider the scenario in which a population of drug-sensitive cancer cells is placed under therapy, leading to a sustained overall decline in tumor size. During this treatment, the cancer cell population may escape extinction via the emergence of mutations that alter a cell's responsiveness to treatment and thus confer a random fitness advantage to the cell under therapy. The stochasticity of the fitness gain in our model reflects the possibility of a spectrum of resistance mutations for any given therapy, or the possibility for a single genetic event to give rise to variable fitness effects within the population.

The sensitive cell population is modeled as binary branching process, 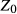, with birth rate 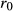 and death rate 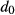. Consider a starting population of 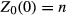 drug-sensitive cells; as the population is undergoing therapy, these cells have a net negative growth rate (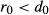). During every birth, there is a probability of 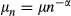 of a mutant drug-resistant offspring with a random, net positive growth rate. Thus, the net growth rate of the sensitive cell population is 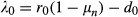; in the following, we denote 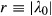. Although this phenotypic variability may be caused my mechanisms other than point mutations, for simplicity we will abuse terminology and refer to the parameter *μ* as a ‘mutation rate’ throughout. The net growth rate of the mutant, 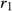, is drawn from a probability density function describing the mutational fitness landscape, *g*(*x*), and the death rate of the mutant is denoted to be 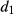. We assume that the fitness landscape *g*(*x*)>0 in an interval [0,*b*] for some finite endpoint *b* and zero otherwise, because cells cannot divide at unbounded rates. The heterogeneous mutant population at time *t* is denoted by 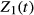 and represents the drug-resistant tumor outgrowth population. In Rebound growth kinetics section, we generalize the model to consider a mixture of sensitive and resistant cells at the start of treatment. In addition, we consider the impact of heterogeneity in the sensitive cell population on recurrence dynamics in the Supplementary Information.

The parameter *α* determines the balance between initial population size of the tumor at the start of treatment and the mutation rate. In the regime *α*<1, in the large *n* limit the probability that resistance arises before the eradication of sensitive cells is 1. This can be seen by considering the mean number of mutations produced by the resistant cell population by time *t*, that is, approximately 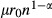. If *α*<1, this grows to infinity, and we are guaranteed a successful resistance mutation prior to extinction. If *α*>1, the mean number of mutations and thus the likelihood of a successful resistance mutation by extinction time tends to 0. In the current work, we are interested in studying the dynamics of recurrent tumors after the development of resistance; therefore, we restrict ourselves to the parameter regime *α*<1.

We next discuss relevant parameter ranges of the initial population size *n* and the mutation rate 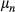 in our model. A solid tumor of diameter 1 cm has been estimated to have approximately 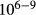 cells, depending on tumor type (James et al. 1999; Detterbeck and Gibson 2008). Thus, considering tumors of diameter *O*(1)−*O*(10) cm, we can obtain estimates of up to 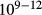 cells. These order of magnitude estimates are also clinically relevant in blood cancers (Sekeres et al. 2007). In some situations, we may be interested in restricting our study to only a small subpopulation of the tumor that is capable of self-renewing and important in driving cancer progression. These small subpopulations, called cancer stem cells, are estimated to be present in frequencies of 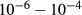 of the total tumor burden (Reya et al. 2001; Sarry et al. 2011). Thus, lower estimates for relevant values of *n* could be within the regime of 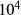, depending on the tumor type and subpopulation of interest. For example, in studies of chronic myeloid leukemia, it has been estimated that 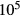 leukemic stem cells are present at the time of diagnosis (Holyoake et al. 1999).

In contrast to cell numbers, the mutation rate parameter 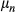 in our model is relatively difficult to quantify, as it represents the rate of resistant cells arising from the sensitive cell population. If we are considering specific point mutations or single base pair substitution rates per cell divisions, various estimates can be obtained in the literature (e.g., 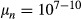; Seshadri et al. 1987; Araten et al. 2005). However, various processes within cells such as chromosomal instability and chromatin reorganization can impact mutation rates, and drug resistance may arise through other types of mechanisms. Thus, in this work we study the behavior of our model as a function of the parameter 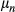.

**Figure 1:**
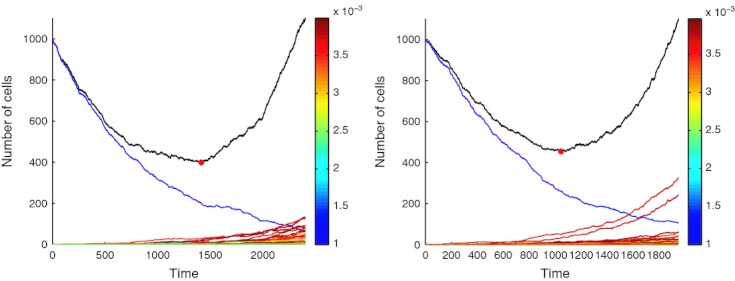
Example simulations of the model demonstrating tumor population trajectories during treatment. The black line indicates the size of the total tumor population, the blue line indicates the initial sensitive population. The multicolored lines represent the temporal dynamics of individual resistant clones created via mutation from the sensitive cell population, 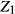 is the sum of these populations. The color of each of these lines is dictated by the clonal fitness (mapped via the colorbar on the right), which is drawn at random from a symmetric beta (2,2) distribution on [0, 0.001]. The red circle in each plot marks the point at which the minimal tumor size is achieved.

Figure 1 shows some sample path simulations of the tumor population over time in this model. As demonstrated, this simple evolutionary model based on exponentially growing branching processes with random growth rates reflects large amounts of variability in rebound tumor composition as well as the timing of recurrence.

## Results

### Rebound growth kinetics

We first investigate the growth kinetics of 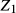, the heterogeneous resistant population driving the cancer recurrence, and its dependence on tumor parameters including the random mutational fitness landscape *g*(*x*). We confine our analysis to the asymptotic regime of large initial tumor size *n*, following the discussion of relevant population sizes in the previous section. In addition, we analyze the dynamics on the sped up time scale of extinction of sensitive cells, that is, *O*( log *n*), since this is the time period during which resistant mutants are produced.

As described previously, each mutation confers a positive fitness advance represented by the random variable *X* ∈ [0,*b*] with probability density function *g*. Define 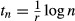 and 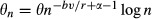. We study the growth kinetics of 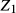 by finding its Laplace transform (LT), given by 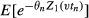, which determines the probability distribution of the 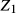 population as a function of time. Using this Laplace transform, we then characterize the behavior of 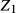 in the large *n* regime.

If 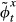 is the Laplace transform of a simple binary branching process with birth rate 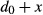 and death rate 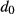, then


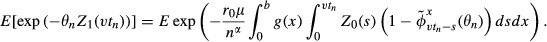


To understand the LT of the limit, it suffices to understand the limit of the expression inside the exponential. As we are considering the large initial population (*n*) limit, we replace 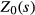 by 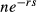:


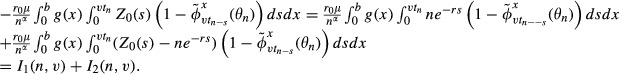


As 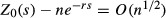, it follows that 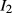 is negligible compared with 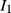. Observe that the actual birth rate of the sensitive cell population is given by 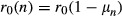. As 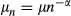, we replace 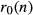 with 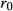. Next recall that (Athreya and Ney 2004)


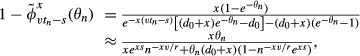
(1)

where the approximation sign is from making the substitution 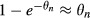. Using this approximation, and the definition of 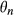, we see that


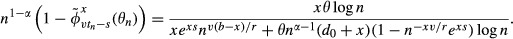


Therefore


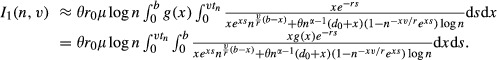


We now consider the integral over *x*. Assume that *h*(·) is a positive decreasing function such that *h*(*n*)→0 and


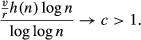


Then,


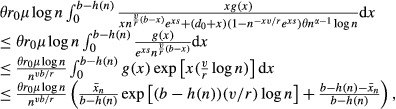


where the final inequality is an application of Bennett's inequality and 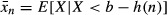. It is easy to see that due to the assumptions on *h*, the last expression goes to 0 as *n*→∞. This argument reveals that mutations only contribute significantly to the overall growth if they confer a fitness close to *b*.

Now consider the contributions from mutations conferring fitness close to *b*,


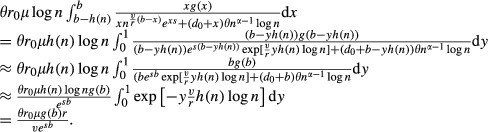


Combining these approximations, we see that


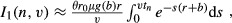
(2)

and therefore for any *θ*>0, 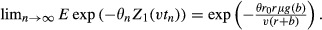 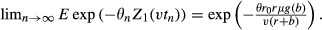 As this is the Laplace transform of a deterministic random variable, we have that for *v*>0,


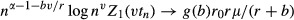


as *n*→∞. Furthermore, based on these results, the 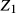 process is approximated by the following:


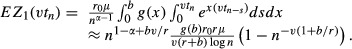
(3)

To understand the dependence of the growth kinetics of 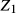, the resistant rebound population, on various model parameters, let us examine the structure of the result in eqn (3). In particular, the term 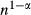 comes from the production of new resistant mutants from the sensitive cell population. The remaining power of *n*, 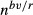 represents the growth of resistant clones. We note that the growth rate of 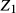 depends on the fitness distribution *g*(*b*) only through its value at the endpoint *b*. In other words, the growth of the population is dominated by the fastest growing mutant in the population, which in our setting *α*<1 is the fittest possible mutant. We also note a delay in the growth rate by the log *n* term in the denominator, which comes from the waiting time needed to achieve a maximally fit mutation. Specifically, to create a mutation with growth rate near *b* we need a large number of mutations, and due to this waiting the maximally fit mutation has a slightly lowered growth rate. The explicit form of this delay is dependent on *n* as the initial population size impacts the chance of developing mutations, and also since the dynamics are analyzed on the time scale of sensitive cell extinction. In particular, a larger *n* implies a faster time scale, so the slowdown is more pronounced. While the growth kinetics of the rebound tumor population depend on the mutational fitness landscape only through its endpoint, as we will show next the diversity of the relapsed tumor depends strongly on the entire shape of this landscape.

Lastly, here we have assumed for simplicity that the sensitive cells are a homogeneous population. While it is likely that the sensitive cell population is already heterogeneous in terms of growth rates by the time a tumor is diagnosed and treated, we show (in the Supplementary material) that the scaling behavior of the resistant population is robust to variation among the decay rates of sensitive cells. We refer the reader to the Supplementary Information for further discussion of this point.

#### Preexisting resistance

An important issue to consider is the presence of preexisting drug-resistant cells (Komarova and Wodarz 2005; Turke et al. 2010; Diaz et al. 2012). Suppose that we decompose the resistant population at time *t* into acquired and preexisting resistant populations as


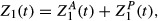


where 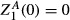 and 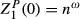 for some *ω* ∈ (0,1). Here, 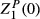 is comprised of a resistant clone with net growth rate *b*. To analyze this new process, we define the following scaling factor for *θ*>0 as


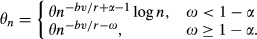


If we consider the resistant population on the approximate time scale of extinction, we see that 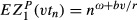 and thus for *ω*<1−*α*


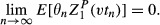


Then, we conclude that if *ω*<1−*α*, the preexisting resistance will have negligible impact on the dynamics of the resistant population in the large *n* regime. In contrast, if *ω*≥1−*α*, we have


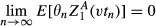


and in this case the acquired resistant population will have a negligible effect on the behavior of the resistant cell population. The distribution of the resistant population as a function of time can be characterized through its Laplace transform as follows:


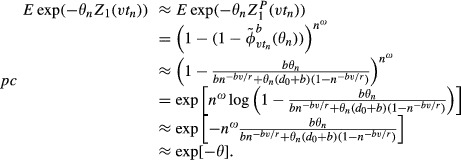


In the previous display, the first equality follows from the independence of the 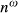 initial preexisting resistant cells, the first approximation follows from (1), and the penultimate approximation from the approximation log (1−*x*)≍−*x* for *x* small. If *ω*≥1−*α*, the preexisting resistant clone will dominate the 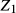 population, and therefore 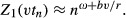


Thus, we have determined conditions under which the level of preexisting resistance will impact recurrence dynamics. In particular, if *ω*≥1−*α*, the relapsed tumor will be largely driven by the initial resistant clone and acquired resistance mutations will not impact tumor growth kinetics significantly. In contrast, when *ω*<1−*α* the resistant population will be largely driven by the creation of a heterogeneous resistant population from mutations acquired during the course of treatment, and the contributions from the preexisting resistant clone will be small in comparison with this population.

### Composition of the recurrent tumor

We next turn our attention to exploring the heterogeneous nature of the recurrent tumor population. To quantify heterogeneity, several measures of diversity are utilized: Simpson's Index, Shannon Index, and species richness. Simpson's Index is defined as the probability that any two randomly selected individuals in the population will be identical, and species richness represents the total number of distinct types in the population. The Shannon Index quantifies the uncertainty in predicting the type of an individual selected at random from the population and is defined mathematically as follows: Suppose 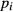, for *i*=1…*N* represents the proportional abundance of the *i*th type in the population. The Shannon Index for this population with *N* types is 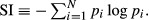


**Figure 2:**
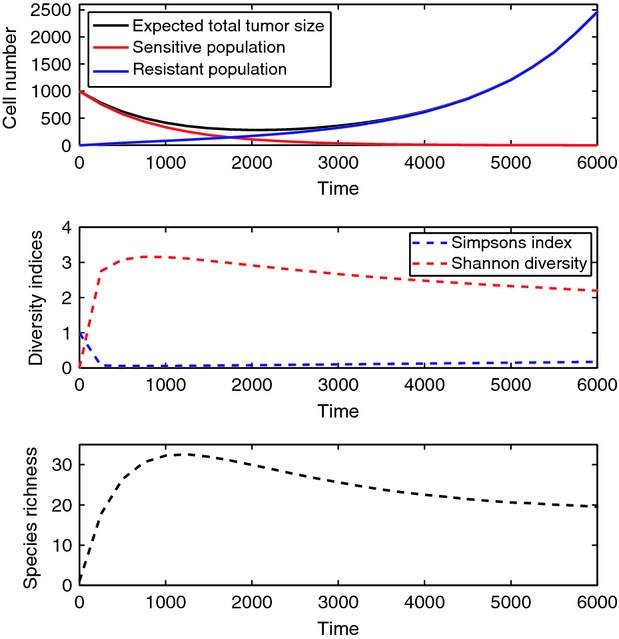
Top: expected population size of tumor, sensitive, and resistant cells versus time, Middle: expected Simpson's and Shannon Index over time. Bottom: expected species richness over time. Parameters: 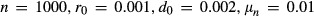. Mutational fitness landscape ∼ Beta(2,2) on [0, 0.001].

We first perform exact stochastic simulations of the model to demonstrate the evolution of these diversity indices over time. Figure 2 demonstrates the evolution of species richness over time as the tumor population declines and rebounds during treatment. We observe that both the Simpsons and Shannon measure of diversity peak during the time period just before tumor recurrence is observed. Then, over time the species diversity decreases and the species richness appears to reach an asymptotic value. This is due to the large production rate of mutants when the sensitive cell population is high, and subsequent extinction of a large fraction of those mutants due to demographic stochasticity. After the sensitive cell population is depleted, no further resistant mutants can be produced, so the surviving escape mutants comprise the rebound tumor population. These dynamics are also reflected in the behavior of the species richness index over time.

**Figure 3:**
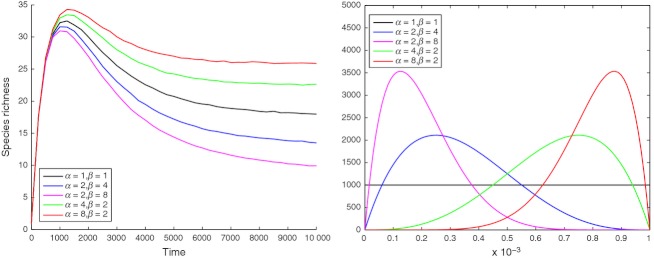
Left: expected species richness versus time for a family of mutational fitness landscapes (beta distribution). Right: corresponding distributions. Parameters: 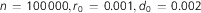.

Figure 3 demonstrates the effect of the mutational fitness distribution on the diversity of the population. In particular, we plot the average species richness in the population over time, for a family of parametrized beta distributions with shape parameters *α* and *β*. Observe that as the mass of *g*(*x*) shifts to the right with increasing shape parameter *α*, the species diversity increases as more of the produced mutants survive. Similarly, as *β* increases the species diversity decreases.

As noted previously, the resistant cell population experiences a large increase in diversity at early times while the sensitive cell population is in its initial decline. However, over time only a fraction of these resistant types produced during this fruitful period survive to become dominant in the relapsed tumor. We next provide analytical estimates of how many of these resistant types produced will emerge as viable subpopulations in the recurrent tumor.

The probability of eventual extinction in a binary branching process with birth rate 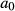 and death rate 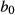 is given by 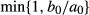. Therefore, the expected number of mutants created by time *t* that will go on to establish viable resistant subpopulations, *S*(*t*) is


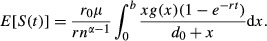


For the case where *g* represents the uniform distribution on [0,*b*],


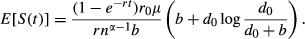


In contrast, the total number of mutant types created by time *t*, *Q*(*t*) has expected value


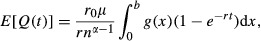


which, in the case of *g* uniform on [0,*b*], takes the form


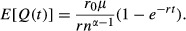


**Figure 4:**
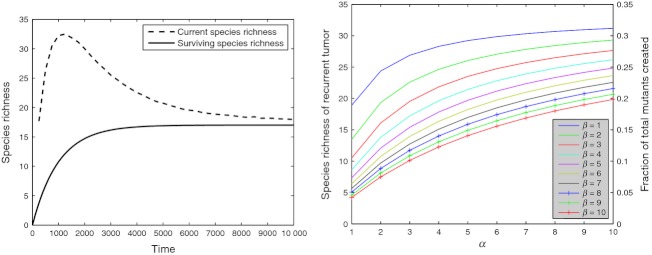
Left: current simulated species richness (dashed) versus time, in comparison with the theoretical estimate of the contribution to permanent species richness (solid). Right: asymptotic species richness of the relapsed tumor for varying mutation fitness landscapes. Parameters: 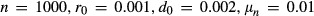.

In the limit as *t*→∞, *E*[*S*(*t*)] represents the asymptotic species richness of the recurrent tumor. Figure 4 (left) demonstrates the convergence of these two quantities: the dashed line denotes the species richness or *extant* number of resistant types in the tumor, obtained through simulation. Since the sensitive cell population is declining exponentially during this time, eventually very few additional resistant types can be produced and overall species richness declines toward an asymptotic value. The solid line represents the theoretical estimate of the number of resistant types present that will eventually be permanently viable in the recurrent tumor. Therefore, this quantity estimates the number of types in the species richness index that will not go extinct and monotonically approaches the asymptotic species richness as new mutants are produced. Thus, these two quantities asymptote to the same value, which represents the overall number of surviving resistant types in the recurrent tumor after the initial transient period. Note that in this plot, the dashed line if extended to the left would reach a species richness of zero at time zero. The plot on the right of Fig. 4 demonstrates the shape of dependence of asymptotic species richness the mutational fitness landscape *g*(*x*). We observe that the diversity of the relapse tumor depends strongly on the shape of the distribution; even when the support of *g*(*x*) is held constant, varying the shape parameters *α* and *β* of the distribution results in species richness varying over the range of 5–30 resistant types in the tumor.

### Recurrence dynamics and tumor composition

Next, we computationally investigate the relationship between the timing of cancer recurrence and the composition of the relapsed tumor. The time of cancer recurrence during therapy represents a measure of the overall survival benefit of therapy. In addition, the size of the tumor at the time of recurrence as well as the timing of recurrence may vary between patients. Stochasticity in the time of recurrence may arise due to variability in the time of emergence of resistant mutants, the fitness of the mutant clones, and the growth dynamics of mutant clones once established. In this section, we are interested in the following question: can we learn clinically useful information about the nature of recurrent tumors, based on clinically observable quantities such as timing of a patient's recurrence and the size of the tumor at recurrence? For example, intuitive assumptions might be that patients who experience later than normal recurrence harbor more indolent resistant clones with lower fitnesses or that a smaller tumor size at recurrence signifies a less diverse relapsed tumor population.

**Figure 5:**
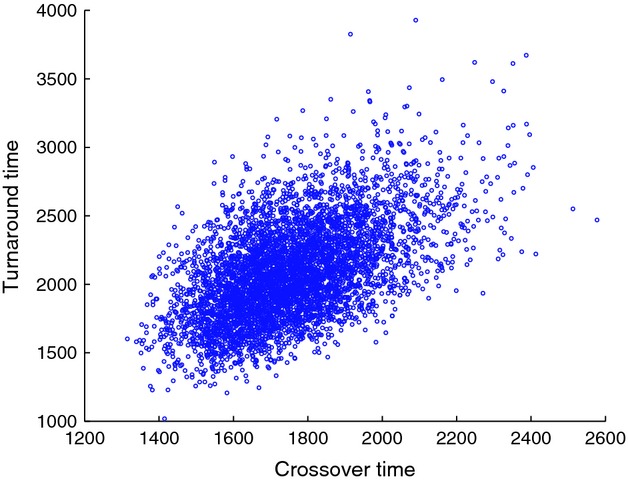
Correlation between the turnaround and crossover times: correlation coefficient 0.54. Parameters: 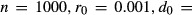 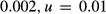. Mutational fitness landscape *U*([0,0.001]).

To investigate this, we consider a tumor consisting initially of sensitive cells, producing resistant mutants with net growth rate drawn from a uniform distribution. We then study statistical correlations between recurrence timing and the diversity of the recurrent tumor. We first consider two stochastic times important to cancer recurrence: (i) the time at which the total population size stops declining and begins to increase, which we denote as the ‘turnaround time’, and (ii) the first time at which the resistant population becomes dominant in the tumor, which we denote as the ‘crossover time’. The turnaround time may be thought of as approximately observable in the clinic, since it is the time at which disease progression is detectable via serial tumor scans. The crossover time is not observable but may be clinically relevant since it can be used to inform optimal times to switch therapies and target another subpopulation within the tumor. Figure 5 shows scatterplots demonstrating that these two stochastic times are strongly correlated. In the following, we utilized just one of these times, the crossover time, as a temporal marker of tumor recurrence to investigate correlations with diversity measures of the tumor. This correlation between turnaround and crossover time is robust to changes in key model parameters such as *α*, which controls the balance between mutation rate and initial tumor size. Thus, in the following investigations, for simplicity we will utilize the crossover time as the marker of recurrence timing.

#### Recurrence timing as a predictor of tumor composition

We first investigate the relationship between recurrence timing and the composition of relapsed tumors. To this end, we calculate diversity measurements of the relapsed tumor (e.g., species richness, Simpson's Index, Shannon diversity) and study the correlations of these measures with recurrence times. To account for a variety of sources of variability in the resistant cell population, we consider a wide spectrum of mutation rates. As we will demonstrate, the system behavior is strongly dependent on this key parameter.

**Figure 6:**
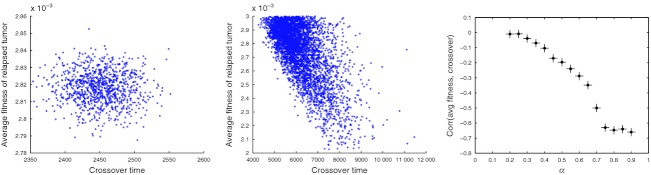
Correlations between the crossover time and the aggressiveness of relapsed tumor. Left: low *α* case: *α*=0.3, correlation coefficient −0.04, and (middle) High *α* case: *α*=0.8, correlation coefficient −0.65, and (right) plot of correlation coefficient between crossover time and average fitness of relapsed tumor, for a spectrum of *α* (mutation rates). Parameters: 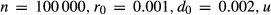 (as dictated by choice of *α*). Mutational fitness landscape *U*([0,0.001]).

Figure 6 exhibits the relationship between the crossover time and the aggressiveness of the relapsed tumor, as indicated by the average growth rate of the resistant cell population, taken at the time when the total tumor size has rebounded to 10% beyond original size of the sensitive tumor. We observe (left panel) that at low values of *α*, which indicates a high mutation rate relative to the tumor size, there is no significant correlation between aggressiveness and recurrence time (correlation coefficient of −0.04). Interestingly, there appears to be a qualitative shift in system behavior at high values of *α* (middle panel), where relapsed tumor aggressiveness is strongly negatively correlated (coefficient of −0.7) to recurrence time. In this regime, late recurrence is indicative of a more indolent tumor on average. Studying this in more detail, we consider a spectrum of mutation rates by varying *α* between 0 and 1 (right panel). We observe a strong dependence of the correlation coefficient on this parameter. In particular, within the regime of high *α* the recurrence time is a good predictor of tumor aggressiveness. For low to moderate values of *α*, there appears to be little value in using recurrence time to predict relapse growth rate.

**Figure 7:**
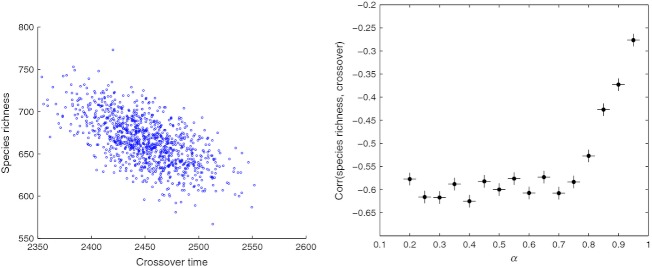
Correlations between the crossover time and the diversity of relapsed tumor. Left: low *α* case: *α*=0.3, correlation coefficient −0.62, and (right) plot of correlation coefficient between crossover time and diversity of relapsed tumor, for a spectrum of *α* (mutation rates). Parameters: 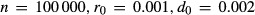 (as dictated by choice of *α*). Mutational fitness landscape *U*([0,0.001]).

The relationship between recurrence timing and the diversity of the relapsed tumor exhibits a similar shift in behavior as *α* is varied. For example, Fig. 7 (left) exhibits a strong negative correlation between the species richness (number of distinct genotypes) of the relapsed tumor and the crossover time, for *α*=0.3. In this case, tumors that recur later than average tend to be more homogeneous than those that recur early. This anticorrelation is also reflected in investigations of the relationship between recurrence time and other measures such as Shannon diversity and Simpson's Index (data not shown). As we increase *α*, we once again observe a qualitative shift in system behavior, as the correlation between recurrence time and diversity is lost at high *α* values (see Fig. 7 right panel). Thus, the crossover time is a good predictor of relapsed tumor diversity in the low to moderate *α* regime, but not in the regime of high *α*.

**Figure 8:**
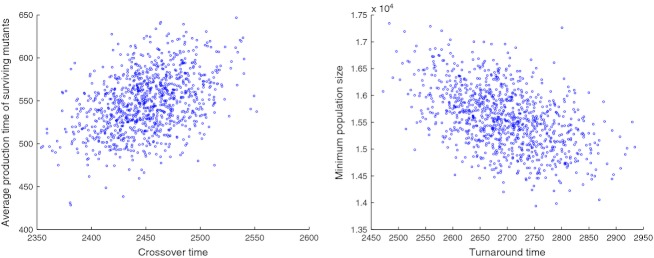
Left: correlation between the crossover time and average production time of surviving mutants in the recurrent population: correlation coefficient 0.42. Right: correlation between recurrence time and the tumor size at the time of recurrence: correlation coefficient −0.48. Parameters: 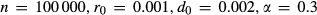. Mutational fitness landscape *U*([0,0.001]).

We next explore the mechanisms causing these observed correlations between recurrence timing, tumor diversity, and aggressiveness. In the low *α* regime, we observe that later recurrence is associated with more homogeneous relapsed tumors, but not associated with tumor aggressiveness. To explain the lack of correlation with tumor aggressiveness, we note that in this regime the mutation production level is high. Thus, it is likely that mutants with near-maximal fitness are produced, and there will be little variation in the average fitness of relapsed tumors between patients. Thus, in this regime, variation in recurrence timing is not driven by differences in tumor aggressiveness. To explain the observed correlation between diversity and recurrence time, we first consider the hypothesis that late-recurring tumors are a result of a lower than normal number of resistant mutants produced, hence leading to lower diversity in the relapse population. Interestingly, an investigation of the relationship between the total number of mutants produced and the recurrence time reveals no such correlation. We next investigate the time at which mutants are produced in the population and find that while there is little correlation between recurrence time and the average time of mutant production, there does exist a correlation with the time of production of the *surviving* mutants in the recurrent population (see Fig. 8 left panel). Since there is relatively little correlation between the number and average time of mutants produced from the sensitive cell population, this indicates that late recurrence occurs due to the death of resistant mutants produced early in the temporal history of treatment.

In contrast, in regimes of high *α*, late recurrence timing is strongly associated with lower tumor aggressiveness. Here, recurrence timing is not strongly correlated with tumor diversity, and variation in recurrence timing is driven by differences in fitness of the mutants produced, rather than in the survival of mutants. To explain these observations, we note that in this regime, mutation production is rare and the fitness of relapsed tumors can vary significantly between patients. Therefore, variability in recurrence time is driven by these differences in fitness, resulting in a strong correlation between late recurrence and lower average fitness. To further support this conclusion, we note that the correlation between the crossover time and the fitness of the most aggressive clone is strong (−0.66) in the high *α* regime, while it is negligible (−0.03) in the low *α* regime. The strong effect of fitness variation on recurrence timing in this regime dominates any effects from stochasticity in survival of these mutants; hence no correlation between diversity and recurrence timing is observed.

**Figure 9:**
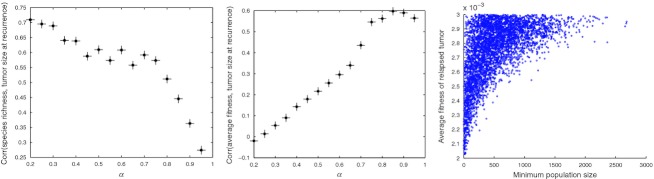
Left: correlation between the tumor size at recurrence and diversity of relapsed tumor, for varying *α*. Middle: correlation between the tumor size at recurrence and average fitness of relapsed tumor, for varying *α*. Right: *α*=0.3, correlation between the minimum population size and the average fitness of the relapsed tumor. Parameters: 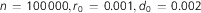. Mutational fitness landscape *U*([0,0.001]).

To summarize, we have observed that as mutation rates are increased, there is a qualitative shift in behavior revealing two distinct regimes. In the regime of low or moderate *α*, the mutant production level is high enough to regularly generate maximally fit mutants. Thus, variability in recurrence timing is more highly associated with demographic stochasticity, or differences in *survival*, of the mutants produced rather than differences in the *production* of mutants. In the regime of high *α*, there is significant variation in the fitness of resistant mutants produced, which strongly influences recurrence timing. Here, variability in recurrence timing is associated with differences in mutant production rather than survival. Lastly, we note that the size of the tumor at the time of recurrence is strongly correlated with the timing of recurrence (see Fig. 8 right panel). Thus, tumor size at recurrence provides similar predictive value for relapsed tumor aggressiveness and diversity, as seen in Fig. 9.

#### Preexisting resistance

We next study the impact of preexisting resistance on the correlations observed in this section. In Model section, we determined analytically that for values of *ω* less than 1−*α*, there will be little impact of preexisting resistance on the relapse dynamics, whereas for *ω* greater than 1−*α*, the relapse tumor will be driven by the preexisting clone. Here, we study the correlation between recurrence time and average fitness of the relapse tumor for varying levels of preexisting resistance and find that the behavior is consistent with results of our earlier analysis. In particular, recall that in the case where *α*=0.3 and the initial population of 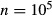 sensitive cells, the correlation coefficient between crossover time and species richness is −0.04 (Fig. 7). If we now include a small population of preexisting resistant cell (*ω*=0.3<1−*α*), the correlation coefficient is −0.04, identical to the case of no preexisting resistance. However, if we consider a larger preexisting resistant population (*ω*=0.8>1−*α*), the correlation coefficient changes significantly to −0.65. This threshold level is dependent on the parameter controlling the balance between mutation rate and initial tumor size, *α*. As this parameter may change between tumor types, therapies, and individual patients, it follows that the threshold frequency determining the impact of preexisting resistance can vary as well. In other words, the same preexisting resistance frequency of *x*% may have negligible effects in one tumor type but strongly impact recurrence dynamics in another tumor type.

#### Connections to clinical data

**Figure 10:**
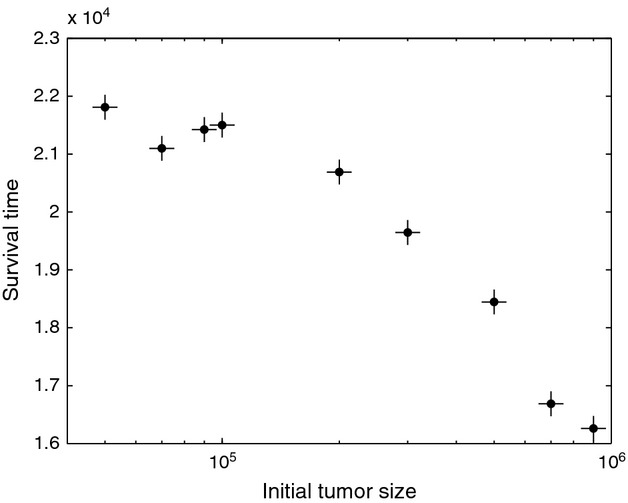
Left: average survival time as a function of initial tumor size. Parameters: 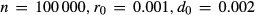. Mutational fitness landscape *U*([0,0.001]).

There have been several clinical studies suggesting that poor prognosis of patients with relapsed disease may be correlated with larger initial tumor size (Port et al. 2003; Mery et al. 2005; Wang et al. 2009). We next utilized our model to investigate this phenomenon. Although the distributions of *in vivo* growth rate parameters for sensitive and resistant cells are generally not available, we are still able to investigate whether these qualitative correlations are predicted by the model by varying parameters. In particular, we first vary the initial population size and study a ‘survival time’, which is defined as the time at which the relapsed tumor reaches twice the initial size (see Fig. 10). We observe that as the initial tumor size increases, the survival time decreases significantly. If we defined the survival time as the time until the relapsed tumor reaches a fixed threshold size, this effect would be even more significant. Thus, we find that, consistent with the trend observed in clinical studies, a larger initial tumor size is correlated with a poorer prognosis.

## Discussion

In this work, we have investigated a model of diversity in relapsed tumors driven by a spectrum of drug-resistance mutations. In particular, we introduced a stochastic branching process model in which an initially declining population can escape certain extinction via the production of mutants whose fitness is drawn at random from a mutational fitness landscape. Using this model, we first applied analytical tools to characterize rebound growth kinetics of the tumor during recurrence. We derived the explicit form of the dependence of the growth kinetics of this population on the initial starting tumor size, mutational fitness landscape, drug response, mutation rate, and growth rates of the sensitive population. In particular, we observed that the exponential growth is dominated by the fittest possible mutant, but there is a correction of log *n* to this growth rate due to the waiting time associated with producing a maximally fit mutant.

We next studied the composition of the relapsed tumor under this model, utilizing ecological measures of diversity such as species richness. We found that while the rebound growth kinetics depend on the mutational fitness landscape only through its value at its endpoint, the diversity of the relapse tumor depends strongly on the full shape of this landscape. We demonstrated that theoretical estimates of the asymptotic species richness matched the asymptotics of the simulated extant species richness in the model. Using these estimates, we demonstrated the variability in asymptotic species richness of the tumor associated with varying the shape parameters of the mutational fitness distribution.

We also computationally investigated the correlations between relapsed tumor diversity and the timing of cancer recurrence. We found that when the mutation rate is high relative to the initial population size, stochasticity in recurrence timing is driven mainly by the random growth and survival of small resistant populations, rather than variability in production of resistance from the sensitive population. Furthermore, late recurrence times are strongly associated with more homogeneous relapse tumors, while early recurrence times are strongly associated with high levels of diversity. In this regime, recurrence timing is not associated with the aggressiveness of the recurrent tumor. In contrast, when the mutation rate is low relative to the initial population size, stochasticity in recurrence timing is driven more by variability in the fitness of resistant mutants produced, rather than their survival. In this regime, a later recurrence time is strongly associated with more indolent tumors, and not associated with the diversity of the relapsed tumor.

The existence of different paradigms of behavior suggests that determining the parameter regime relevant for specific tumor types and resistance mechanisms (e.g., point mutations, epigenetic alterations, amplifications) is an important factor in utilizing recurrence time or size of the tumor at relapse as predictive tools for estimating the aggressiveness or diversity of relapsed tumors. For example, consider the scenario of emergence of resistance to the tyrosine kinase inhibitor erlotinib during treatment of non-small cell lung cancer (NSCLC). Here, we estimate that the size of a NSCLC tumor lies in the range 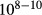 (where a 1 cm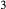 tumor is approximately 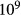 cells; Talmadge 2007). The T790M point mutation in the EGFR kinase domain has been implicated in the development of resistance to this drug (Pao et al. 2004). If we assume an initial population size of 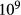, and consider relapse due to point mutations occurring at an estimated rate of 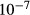 or 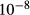, we are likely to be in a high *α* regime. Thus, we would expect the recurrence time (or tumor size at recurrence) to be indicative of the aggressiveness of the tumor. Although we are not aware of any clinical studies of this nature in NSCLC, this phenomenon has been observed in a glial brain tumor study, which concluded that a decreased time to tumor recurrence is associated with a more aggressive phenotype, as indicated by higher levels of hypoxia detected (Evans and et al. 2004). However, in a tumor type where alterations causing a resistant phenotype occur at a higher rate (e.g., in the presence of chromosomal instability), we may expect behavior in the low *α* regime, where no correlation exists between tumor size at recurrence and aggressiveness. This may be the case, for example, in the case of chronic myeloid leukemia where mutation rates are elevated by the BCR-ABL mutation or in colon carcinogenesis where somatic deletions in simple repeat sequences have been shown to increase mutation rates in these tumors (Ionov et al. 1993).

We also considered the impact of heterogeneity of the initial population on these findings. In particular, we first studied the impact of preexisting resistant cells on recurrence dynamics. We analytically derived simple conditions on the relationship between the initial size, mutation rate, and preexisting resistant population size that can be used to determine whether preexisting resistance plays a significant role in the relapsed tumor. Although the initial frequency of resistant cells can be difficult to determine clinically, especially in cases where the mechanism of resistance is unknown, our results could be used to help determine the presence or absence of a substantial clone of preexisting resistance based on clinical observations. For example, we have shown that in the low *α* regime, if the initial population of resistant cells is negligible, there should be no correlation between the size of the tumor at relapse (or recurrence time) and the aggressiveness of the tumor. Thus, if clinical observations do reveal a strong correlation between tumor size at recurrence and aggressiveness, this may suggest that a substantial preexisting resistant population was present at the start of therapy. In addition, we noted that the threshold level for determining the impact of preexisting resistance on recurrence dynamics is strongly dependent on *α*, the parameter controlling the balance between mutation rate and initial tumor size. This parameter may vary significantly between tumor types, therapies, and individual patients. Therefore, the same level of preexisting resistance may have negligible effects in one tumor type or individual but strongly impact recurrence dynamics in another.

These findings also provide us with some insight into the clinical treatment and prognosis of relapsed or recurrent tumors. For example, if certain tumor types are known to be in the low *α* regime, patients who progress rapidly after an initial response to therapy may benefit more from combination therapies to combat high levels of heterogeneity in their recurrent tumors, while patients who progress late can be expected to harbor less clones. Also, for patients whose tumor types are known to be in a high *α* regime, a late relapse can be given a better prognosis from the time of recurrence. Furthermore, a detailed quantitative understanding of how initial tumor size/composition, mutation rates, and growth kinetics conspire to drive recurrence dynamics, and the composition of relapsed tumors can be eventually utilized to design treatment schedules tailored according to patient, tumor type and size, and drug. However, to bridge the gap between these theoretical predictions and clinical recommendations, substantial more effort must be made in (i) experimental identification of model parameters (which would identify the relevant regime for each tumor type and drug combinations) and (ii) model validation through experiments and detailed clinical data analysis of tumor evolution *in vivo*. In the following, we discuss the recent development of novel experimental techniques that may be used to carry out these goals.

Our studies have quantified the impact of the mutational fitness landscape on the composition of recurrent tumors and underscore the importance of experimental efforts to quantify mutation rates and the distribution of random fitness effects of mutations in cancer. Quantification of these parameters has been largely elusive due to experimental limitations, despite our recognition of their importance in understanding tumor evolution. However, currently many single-cell analysis platforms are being developed to quantify the heterogeneity in cell populations. These technologies include microfluidics systems, such as the microscale cantilever described in (Son et al. 2012), which is capable of measuring single-cell mass changes as a function of cell cycle progression, and high-content automated imaging systems, which are being used to quantify phenotypic variability (i.e., growth rate, migration, etc.) between individual cells (Quaranta et al. 2009). These novel and powerful experimental techniques can be utilized to determine fitness distributions of growth rate changes conferred by specific mutations under a variety of environmental conditions. The availability of such data in the future will be instrumental in making clinical predictions using evolutionary models of tumor progression.

Clinical and experimental validation of model predictions of relapsed tumor composition over time and recurrence timing are important for correct calibration and refinement of our model. However, intratumoral heterogeneity is traditionally difficult to dynamically quantify *in vivo*. Recently, there has been renewed interest in the impact of tumor heterogeneity and adaptation on patient outcome (Gerlinger et al. 2012). For this reason, significant emphasis has been placed on the development of tools to globally assess the dynamic state of a tumor (i.e., changes in tumor complexity and composition) rather than single snapshots that fail to capture the overall tumor behavior. Circulating tumor DNA, serum protein biomarkers, and circulating tumor cells are a few of these promising noninvasive diagnostic tools being used to monitor disease progression (Taniguchi et al. 2011; van de Stolpe et al. 2011). A recent study by Diaz et al. (2012) demonstrated the utility of circulating tumor DNA in identifying and tracking the levels of rare mutant KRAS alleles throughout the course of treatment in 28 colorectal cancer patients using serial serum sampling. Thus, noninvasive techniques for the quantification of the evolution of heterogeneous tumor cell populations over time are now becoming more widely available.

Ultimately, tumors are complex adaptive systems that should not be evaluated as static objects. Our evolutionary modeling has provided insights into the factors driving the timing and composition of recurrent tumors and how clinically observable factors such as recurrence time or size may reveal useful information about the composition of relapsed tumors. In conjunction with recent novel experimental advancements in single-cell profiling and dynamic tumor profiling, we believe these results may contribute to a better understanding of tumor recurrence and eventually help in guiding treatment decisions in the clinic.

## Supporting Information

**Data S1.** Analysis of the effects of heterogeneity in the sensitive cell population on rebound tumor growth kinetics.
