# Supplementary material for: Cancer as a moving target: understanding the composition and rebound growth kinetics of recurrent tumors
Source: Evol Appl. 2012 Nov 16;6(1):54–69. doi: 10.1111/eva.12019 (PMC3567471; doi:10.1111/eva.12019)
Supplement: Supplementary file 1 [file eva0006-0054-SD1.pdf]

4 Supplementary Information

In this section we investigate the effects of a heterogeneous sensitive cell population. Specifically assume that the sensitive cell population at time t is

$$Z_0(t) = \sum_{j=1}^{N} Z_{0,j}(t),$$

where for each j, $Z_{0,j}$ is a sub-critical binary branching process with birth rate r_0, death rate $d(j)$ and net decay rate $r(j) = |r_0 - d(j)|$. Lastly the initial conditions are given by $Z_{0,j}(0) = p_j n$ where $p_j \in (0,1)$. Define $r = \min\{r(j) : 1 \leq j \leq N\}$, then define $t_n = \frac{1}{r}\log n$ and for $\theta > 0$

$$\theta_n = n^{-bv/r+\alpha-1}\log n.$$

We can then write the Laplace transform of the rescaled and sped up resistant cell process as

$$\begin{aligned} E\exp\left(-\theta_n Z_1(vt_n)\right) &= E\exp\left(-\frac{r_0\mu}{n^\alpha}\int_0^b g(x)\int_0^{vt_n}\left(1-\tilde{\phi}^x_{vt_n-s}(\theta_n)\right)\sum_{j=1}^{N} Z_{0,j}(s)dsdx\right) \\ &\approx \exp\left(-\frac{r_0\mu}{n^\alpha}\int_0^b g(x)\int_0^{vt_n}\left(1-\tilde{\phi}^x_{vt_n-s}(\theta_n)\right)\sum_{j=1}^{N} e^{-r(j)s}p_j n dsdx\right), \end{aligned}$$

where we replace Z_0 by its mean. Define

$$\tilde{I}_1(n,v) = r_0\mu n^{1-\alpha}\int_0^b g(x)\int_0^{vt_n}\left(1-\tilde{\phi}^x_{vt_n-s}(\theta_n)\right)\sum_{j=1}^{N} e^{-r(j)s}p_j dsdx$$

Then note that the derivation for the approximation of $I_1(n,v)$ in (2) is independent of the value of r in the exponent in the integrand, and therefore

$$\tilde{I}_1(n,v) \approx \frac{\theta r_o \mu r g(b)}{v}\sum_{j=1}^{N}\frac{p_j}{r(j)+b},$$

which gives us the approximation

$$Z_1(vt_n) \approx n^{1-\alpha+bv/r}\frac{r_o\mu r g(b)}{v}\sum_{j=1}^{N}\frac{p_j}{r(j)+b}.$$

Comparing with the approximation in the setting of homogenous sensitive cell population we see only a change in the values of the constant.
