# Supplementary figures and images for: Cancer as a moving target: understanding the composition and rebound growth kinetics of recurrent tumors
Source: Evol Appl. 2012 Nov 16;6(1):54–69. doi: 10.1111/eva.12019 (PMC3567471; doi:10.1111/eva.12019)

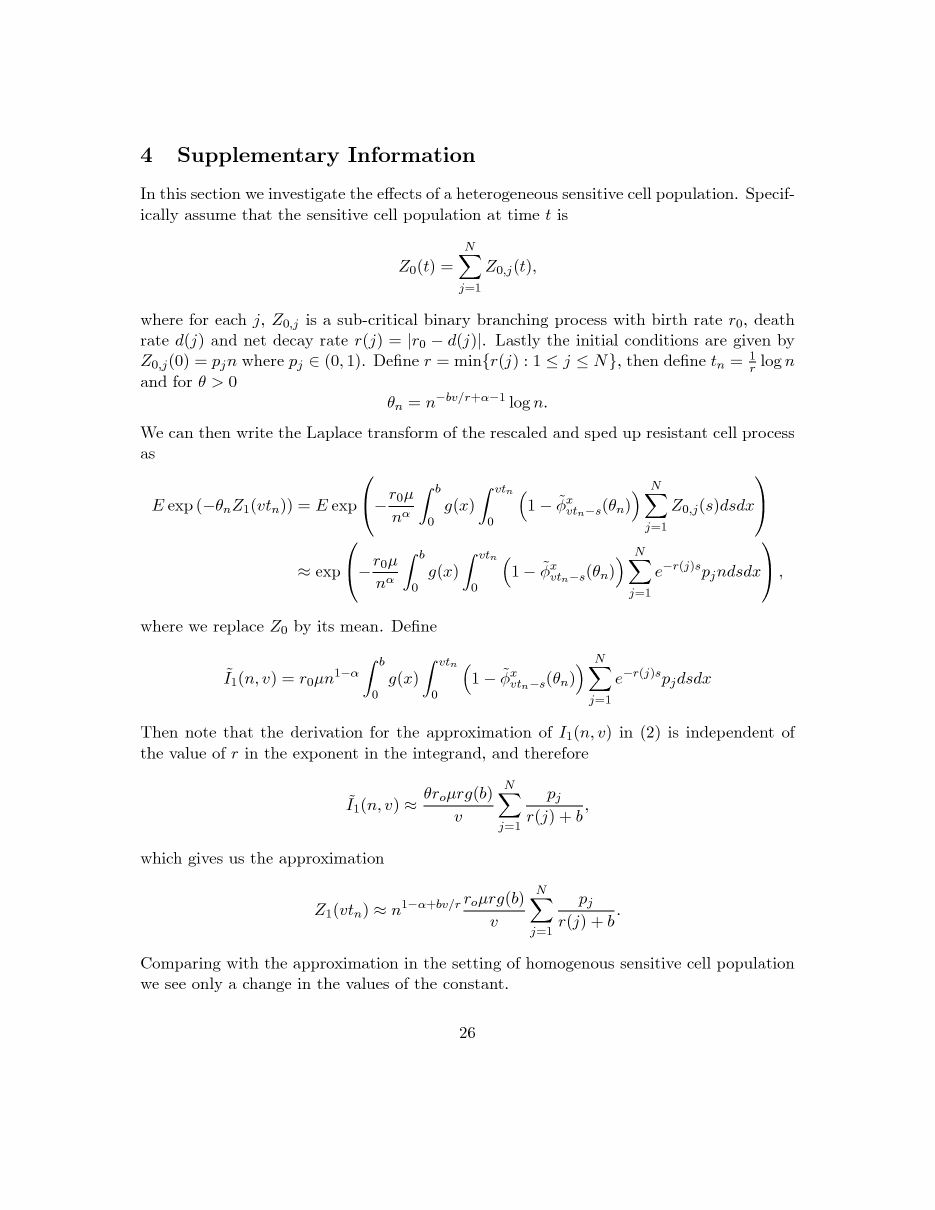

Supplement: Supplementary file 2 [file eva0006-0054-SD2.png]
